# Bovine Respiratory Syncytial Virus Enhances the Adherence of *Pasteurella multocida* to Bovine Lower Respiratory Tract Epithelial Cells by Upregulating the Platelet-Activating Factor Receptor

**DOI:** 10.3389/fmicb.2020.01676

**Published:** 2020-07-31

**Authors:** Putu Eka Sudaryatma, Akatsuki Saito, Hirohisa Mekata, Meiko Kubo, Watcharapong Fahkrajang, Eugene Mazimpaka, Tamaki Okabayashi

**Affiliations:** ^1^Graduate School of Medicine and Veterinary Medicine, University of Miyazaki, Miyazaki, Japan; ^2^Department of Veterinary Science, Faculty of Agriculture, University of Miyazaki, Miyazaki, Japan; ^3^Center for Animal Disease Control, University of Miyazaki, Miyazaki, Japan; ^4^Organization for Promotion of Tenure Track, University of Miyazaki, Miyazaki, Japan; ^5^Takazaki Meat Inspection Center, Miyazaki, Japan

**Keywords:** platelet-activating factor receptor, BRSV, bovine respiratory epithelial cells, surface receptor, coinfection, pneumonia, BRDC, *Pasteurella multocida*

## Abstract

Coinfection by bovine respiratory syncytial virus (BRSV) and *Pasteurella multocida* (PM) frequently has been observed in cattle that develop severe pneumonia. We recently reported that BRSV infection significantly increased PM adherence to bovine lower respiratory tract epithelial cells. However, the molecular mechanisms of enhanced PM adherence are not completely understood. To investigate whether BRSV infection regulates any cellular adherence receptors on bovine bronchus- and lung-epithelial cells, we performed proteomic and functional analyses. The proteomic analysis showed that BRSV infection increased the accumulation of the platelet-activating factor receptor (PAFR) in both cell types. Molecular experiments, including specific blockade, knockdown, and overexpression of PAFR, indicated that PM adherence to these cell types depended on PAFR expression. These findings highlight the role, in cattle with severe pneumonia, of the synergistic effect of coinfection by BRSV and PM in the lower respiratory tract.

## Introduction

The bovine respiratory disease complex (BRDC) is the most common health condition affecting feedlot and dairy cattle production (Gershwin et al., 2015). The disease has been proposed to result from interactions among multiple viral and/or bacterial pathogens (Larsen et al., 2001; Kalina et al., 2006; Blodörn et al., 2015). Bovine respiratory syncytial virus (BRSV), an enveloped negative-stranded RNA virus of the genus *Orthopneumovirus* and family *Pneumoviridae* (Spilki et al., 2006), shows a close genetic relationship with human respiratory syncytial virus (HRSV) (Valarcher et al., 2000). BRSV is recognized as one of the most important viral agents of BRDC, causing lower respiratory tract and lung infections in beef and dairy calves (Larsen et al., 2001; Beaudeau et al., 2010). BRSV infections in cattle augment the number of adherent bacteria (Tjønehøj et al., 2003; Tizioto et al., 2015) by destroying the normal transport mechanism used by airway epithelial cells, allowing the flow of bacteria through the lower airway and lung (Spilki et al., 2006; Jordan et al., 2015). Furthermore, BRSV infection of the lower respiratory tract can stimulate a substantial increase in bacterial load and lead to secondary complications and the development of severe pneumonia (Johnston et al., 2019).

In humans, infection by respiratory viruses (e.g., HRSV, coronavirus, and influenza A virus) regulates the expression of cellular receptors such as intercellular adhesion molecule-1 (ICAM-1), carcinoembryonic antigen-related cell adhesion molecule, fibronectin, and platelet-activating factor receptor (PAFR) (Garcia et al., 2010; Golda et al., 2011; Hendricks et al., 2016). Those studies suggested that upregulation of cellular receptors following virus infection facilitates bacterial adherence to respiratory epithelial cells (Hament et al., 2004; Avadhanula et al., 2006; Wang et al., 2009). PAFR in virus-infected respiratory epithelial cells is used as a recognition molecule for respiratory bacteria such as *Streptococcus pneumoniae*, *Haemophilus influenzae*, and *Pseudomonas aeruginosa* (Hament et al., 2004; Yokota et al., 2012; Bellinghausen et al., 2016). Thus, previous work has suggested that virus infection plays an important role in inducing the production of adhesion molecules on the respiratory epithelial cells, facilitating bacterial adherence.

In our previous studies, we demonstrated that BRSV regulates adherence of *Pasteurella multocida* (PM) to human and bovine respiratory epithelial cells (BRECs). Specifically, we showed that pre-infection of bovine bronchus epithelial cells (bBECs) and lung epithelial cells (bLECs) with BRSV increased adherence by PM; this effect was not seen for bovine trachea epithelial cells (bTECs) (Sudaryatma et al., 2019). Those results indicated that BRSV may reproduce better in the lower respiratory tract, leading in turn to increased adherence of bacteria. Notably, BRSV infection appears to facilitate bacterial adherence by modulating the expression of surface proteins on BRECs. To our knowledge, only a few studies have examined viral induction of changes in BRECs, despite the fact that cells of this type are the portals of entry for both viruses (Goris et al., 2009; Eberle et al., 2016) and bacteria (Cozens et al., 2019).

Based on these results, we suspected that BRSV infection of lower BRECs induces the production of cellular receptors and that these receptors are used as adhesion molecules by PM, increasing the risk of development of severe pneumonia. To clarify the role of BRSV infection in modulating PM adhesion to BRECs, we sought to identify the unknown surface receptor(s) whose expression is altered in cells infected by BRSV, thereby facilitating the adherence of PM.

## Materials and Methods

### Cells

Bovine respiratory epithelial cells were collected from three freshly slaughtered adult Japanese black cattle, as described previously (Sudaryatma et al., 2019). Briefly, epithelial cells were recovered from organ trachea (bTECs), bronchus (bBECs), and lung (bLECs). Cells were maintained in Dulbecco’s modified Eagle’s medium/Nutrient Mixture F-12 GlutaMAX (DMEM/F12; Thermo Fisher Scientific, MA, United States) supplemented with 2% heat-inactivated fetal bovine serum (FBS; Biowest, France), 100 U/ml penicillin, 100 mg/ml streptomycin, 1 μg/ml amphotericin-B, 10 ng/ml epidermal growth factor, 1% insulin–transferrin–selenium, 1% non-essential amino acids, and 2 mM L-glutamine (all obtained from Wako, Japan). HEK293T cells were maintained in Dulbecco’s modified Eagle’s medium (DMEM; Nacalai Tesque, Japan) supplemented with 10% FBS, 100 U/ml penicillin, and 100 mg/ml streptomycin.

### Virus and Bacterium

Bovine respiratory syncytial virus strain 2205027-1 and *P. multocida* strain 2368 were prepared as described previously (Sudaryatma et al., 2019). For infection, virus or bacteria were diluted in antibiotic- and serum-free DMEM/F12 to achieve the desired multiplicity of infection target. A BRSV inoculum that would generate multiplicity of infection (MOI) 1 was subjected to ultraviolet light illumination for 1 h to inactivate the virus (UV-BRSV). The inactivation was confirmed by plaque assay as described previously (Sudaryatma et al., 2018).

### BRSV Infection of BRECs

Bovine respiratory epithelial cells were infected with BRSV as described previously (Sudaryatma et al., 2019). Briefly, epithelial cells from trachea (bTECs), bronchus (bBECs), and lung (bLECs) were seeded overnight and infected with infectious BRSV (MOI 0.1 or MOI 1) or UV-BRSV. Cells were washed three times with phosphate-buffered saline (PBS) and incubated for 3 days in a complete culture medium for further experiments.

## Proteomic Analysis

Cell lysates of BRSV-infected or uninfected BRECs were fixed with methanol. Analysis by liquid chromatography was performed using UltiMate 3000 RSLCnano (Thermo Fisher Scientific) and a Q Exactive (Thermo Fisher Scientific). Proteins were classified as positively detected if peaks were identified in 2 out of 3 treatment groups. Protein analysis was performed using Proteome Discoverer 2.2. The Venn diagrams were designed using the InteractiVenn software^1^ (Heberle et al., 2015). The differentially expressed proteins were defined as those exhibiting a ratio larger than 1.5 with a *p*-value cutoff <0.05.

### Transient Silencing of PAFR

Gene silencing of the bovine *PAFR* genes in BRECs was performed with Lipofectamine RNAiMAX transfection reagent (Thermo Fisher Scientific). Two pairs of stealth small-interfering RNAs (siRNAs) targeting the bovine PAFR genes were purchased from Thermo Fisher Scientific. The strand-paired PAFR1 sequences were 5′-CGUGUGGACUCAGAGUUCCGAUACA-3′ (sense) and 5′-UGUAUCGGAACUCUGAGUCCACACG-3′ (antisense); the strand-paired PAFR2 sequences were 5′-ACAUCACACGCUGCUUUGAACAUUA-3′ (sense) and 5′-UA AUGUUCAAAGCAGCGUGUGAUGU-3′ (antisense). Disso- ciated cells were added to a mixture of 30 pmol siRNA and 9 μl of Lipofectamine RNAiMAX Reagent (Thermo Fisher Scientific) in 500 μl Opti-MEM (Thermo Fisher Scientific). The siRNA Negative Control of Stealth RNAi (Thermo Fisher Scientific) was used as a non-targeting control in these experiments. At 24 h after transfection, cells were infected with BRSV (MOI 1) and maintained for 3 days for further experiments.

### Quantification of Viral RNA in BRECs

Quantitative real-time RT-PCR assays (qRT-PCR) were performed as described previously (Sudaryatma et al., 2019). Briefly, viral RNA in the culture supernatant of BRSV-infected cells was extracted using the NucleoSpin Virus Kit (TaKaRa Bio, Japan) according to the manufacturer’s protocol. qRT-PCR was performed with the One Step PrimeScript RT-PCR Kit (Perfect Real Time) (TaKaRa Bio) on a LightCycler 96 system (Roche, CA, Untied States).

### Construction of a Plasmid Vector Expressing Bovine PAFR

Mammalian expression vectors for bovine PAFR (pCEP4-bPAFR) and EGFP (pCEP4-EGFP) were constructed for use in this work. A cDNA of *PAFR* was prepared from bLECs by two-step RT-PCR using SuperScript IV Reverse Transcriptase (Thermo Fisher Scientific) and PrimeSTAR GXL DNA Polymerase (TaKaRa Bio). A cDNA of *EGFP* was amplified from the pEGFP plasmid (Clontech, Japan). The gene-specific primers for amplification of *PAFR* were *Not*I-bPAFR-F (5′-TCGAGCGGCCGCGCCACCATGGAGCCAAATAATTCCTTT C-3′) and bPAFR-*Bam*HI-R (5′-GCTCGGATCCCTAATATT TGAGGGATTTGACAGGG-3′); those for EGFP were *Not*I-Kozak-EGFP-F (5′-GCGGCCGCGCCACCATGGTGAGCAAG GGCGAGGA-3′) and EGFP-*Bam*HI-R (5′-GGATCCTTACT TGTACAGCTCGTCCATGC-3′). The amplified PCR products were digested with *Not*I and *Bam*HI. The digested fragments were purified and ligated into a similarly digested pCEP4 vector (Thermo Fisher Scientific) using a Mighty Mix DNA Ligation Kit (TaKaRa Bio). The reaction mixture was used for transformation of NEB 5-alpha F’I^*q*^ Competent *Escherichia coli* (High Efficiency) (NEB, Japan) according to the manufacturer’s protocol. *E. coli* harboring these plasmids were selected on Luria–Bertani (LB) agar containing 50 μg/ml ampicillin. Resulting colonies were picked to LB broth containing ampicillin and grown overnight at 37°C with shaking, and plasmids were purified using the PureYield Plasmid Maxiprep System (Promega, Japan) according to the manufacturer’s protocol. The PCR-amplified regions were verified by sequencing on an Applied Biosystems 3130xl Genetic Analyzer (Thermo Fisher Scientific).

### Overexpression of PAFR

A range of amounts of the pCEP4-bPAFR plasmid was used for transfection. The pCEP4-empty plasmid was used to adjust the total amount of DNA to 1,000 ng. All plasmids were diluted in Opti-MEM and transfected into HEK293T cells (3 × 10^5^ cells/well in a 12-well plate) using the TransIT-LT1 transfection reagent (TaKaRa Bio). Cells were maintained for 1 day for further experiments. Transfection efficiency was confirmed by counting the number of EGFP-positive cells via flow cytometry using a BD FACSCanto II (BD Biosciences, CA, United States). EGFP expression demonstrated that the transfection efficiency exceeded 60%.

### Specific Blockade of PAFR With an Antibody and an Antagonist

Blocking of bovine PAFR assay was performed using either an anti-PAFR antibody (ab104162; Abcam, Japan) or a PAFR antagonist (ginkgolide B; Selleckchem, Japan). BRSV-infected cells were treated with serially diluted antibody or ginkgolide B for 1 h at 37°C. Normal rabbit serum or 0.001% DMSO served as a negative control, respectively. In pilot experiments, we determined that ginkgolide B concentrations of 118.73 and 36.83 μM were safe (non-cytotoxic) for BRECs or HEK293T cells (respectively) under our culture conditions.

### Western Blot Analysis

The BRECs or HEK293T cells grown in 6-well plates were lysed by resuspension in 300 μl of lysis buffer (150 mM NaCl, 50 mM Tris–HCl, pH 7.6, 1% NP-40, protease inhibitor, and phosphatase inhibitor) followed by three rounds of 10-s sonication. The protein concentrations of each sample were determined using a bicinchoninic acid protein assay reagent kit (TaKaRa Bio). Aliquots of 10 μg of protein per lane for each sample were electrophoretically separated in a 4–15% sodium dodecyl sulfate-polyacrylamide gel and transferred to a polyvinylidene difluoride membrane using the Trans-Blot Semi-Dry Electrophoretic Transfer Cell (all from Bio-Rad Laboratories, CA, Untied States). The membranes were saturated overnight at 4°C with blocking buffer (25 mM Tris, pH 8.0, 125 mM NaCl, 0.01% Tween-20, and 3% bovine serum albumin). The membranes then were incubated with either rabbit anti-PAFR (#ab104162; Abcam, Japan) or mouse anti-glyceraldehyde 3-phosphate dehydrogenase (GAPDH) (#ab9482; Abcam) primary antibodies at room temperature for 1 h. After vigorous washing, the membranes were incubated with a secondary goat anti-rabbit IgG antibody conjugated with horseradish peroxidase (#ab205718; Abcam) at room temperature for 1 h. The signals were developed by the Western BLoT Hyper HRP (TaKaRa Bio) reagent and detected with the ChemiDoc Touch Imaging System (TaKaRa Bio).

### Bacterial Adherence Assay

Cells infected with either infectious BRSV or UV-BRSV, or cells treated with antibody, siRNA, or ginkgolide B, were exposed to PM (MOI 100) for 1 h. In this assay, the numbers of colony-associated bacteria were normalized to the numbers of epithelial cells as described previously (Sudaryatma et al., 2019). For the fluorescence assay, PM was labeled with fluorescein isothiocyanate (FITC) (Dojindo, Japan) as described previously (Sudaryatma et al., 2018). The BRSV-infected or antibody-treated cells then were exposed to the FITC-labeled PM for 1 h. Adherence of the FITC-labeled PM to the cells was observed using an Olympus DP-74 (Olympus, Japan) confocal fluorescence microscope.

In a flow-cytometry assay, the PAFR-overexpressing HEK293T cells or ginkgolide B-treated cells were exposed to the FITC-labeled PM. Cells were collected and fixed with 4% paraformaldehyde. Fixed cells were incubated with rabbit anti-PAFR at room temperature for 1 h, followed by the DyLight 650-conjugated donkey anti-rabbit IgG secondary antibody (#A120-208D5; Bethyl, TX, United States). Expression of PAFR and the adherence of the FITC-labeled PM were quantified using the BD FACSCanto II.

### Statistical Analysis

All BREC experiments of each organ site were performed individual culture derived from three individual animals. For overexpressing HEK293T cells, three independents cultures were performed for quantification. Data are presented as the mean and standard error (SEM) of triplicate measurements. Statistical significance was assessed using two-tailed one-way analysis of variance (ANOVA) with a *post hoc* Dunnett’s test where significance was indicated. *p-*values < 0.05 were considered significant. Statistical analyses were performed using the RStudio software package, version 1.0.143 (RStudio, 2015).

## Results

### Proteomic Changes in BRECs Infected With BRSV

**FIGURE 1:**
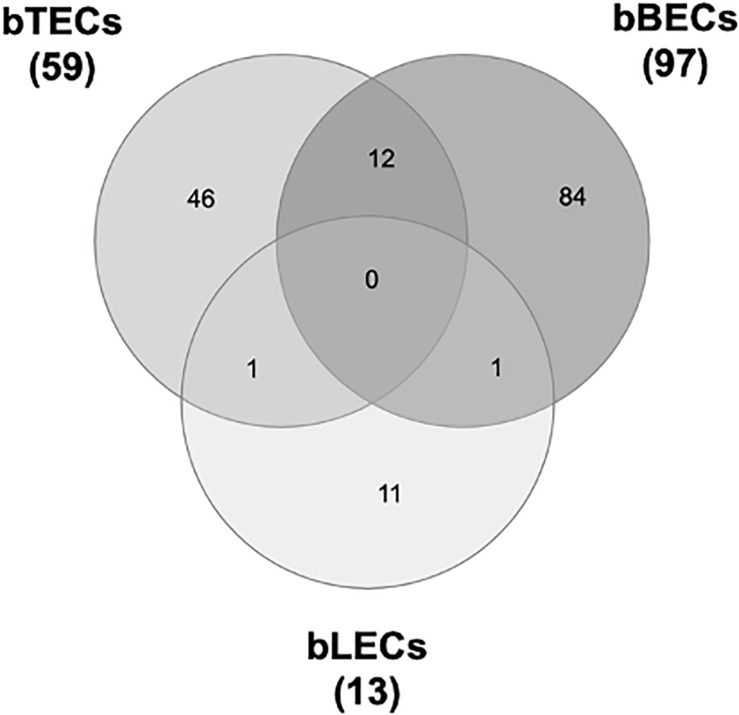
Upregulated proteins in bovine trachea (bTECs), bronchus (bBECs), and lung (bLECs) epithelial cells infected with bovine respiratory syncytial virus (BRSV). Cells were infected with BRSV (multiplicity of infection 1) for 3 days. The overlap in proteins that are significantly upregulated following BRSV infection are shown. Data represent the mean of three independent experiments using cells derived from three different animals.

To identify surface proteins upregulated by BRSV infection, we performed a proteomic analysis of BRECs which revealed that BRSV infection resulted in significant changes in the levels of 59 proteins in bTECs, 97 proteins in bBECs, and 13 proteins in bLECs. Differentially expressed proteins with ratios >1.5-fold compared to an uninfected control were classified as “upregulated proteins.” Gene ontology analysis revealed that the upregulated proteins were involved in the membrane and protein binding pathways, according to the cellular component and molecular function annotations, respectively. Of the upregulated proteins, just one—PAFR—was shared between bBECs and bLECs (Figure 1). The data generated was deposited in the jPOSTrepo (Japan proteome standard repository) (Okuda et al., 2017) member of ProteomeXchange Consortium with dataset identifier JPST000862.

### BRSV Infection Induces PAFR Expression in Bovine Lower Respiratory Epithelial Cells

**FIGURE 2:**
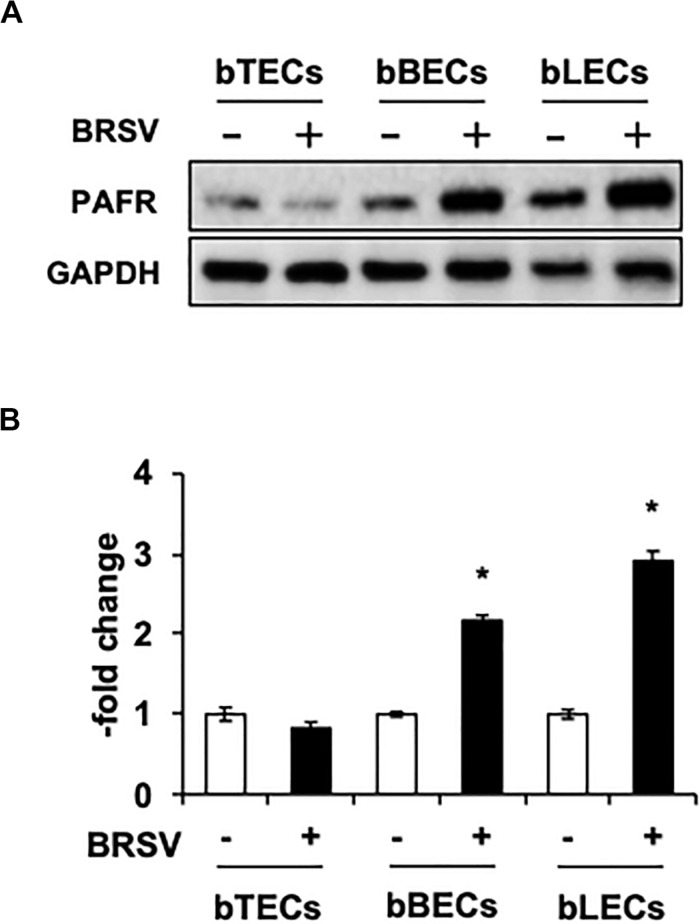
Bovine respiratory syncytial virus (BRSV) infection upregulates platelet-activating factor receptor (PAFR) in bovine respiratory epithelial cells. **(A)** Expression levels of PAFR in BRSV-infected trachea (bTECs), bronchus (bBECs), and lung (bLECs) epithelial cells compared to the respective uninfected cells. Protein expression was evaluated by immunoblotting using the anti-PAFR antibody. **(B)** Levels of PAFR were calculated by normalizing the band density of PAFR by that of a housekeeping protein (glyceraldehyde 3-phosphate dehydrogenase, GAPDH) in the respective sample (lane); values then were normalized to those in uninfected cells. Data in **(B)** represent the mean ± SEM of three independent experiments using cells derived from three independent animals conducted in triplicate. *p < 0.05 for comparison to the respective no-virus control (by two-tailed one-way ANOVA with *post hoc* Dunnett’s test).

**FIGURE 3:**
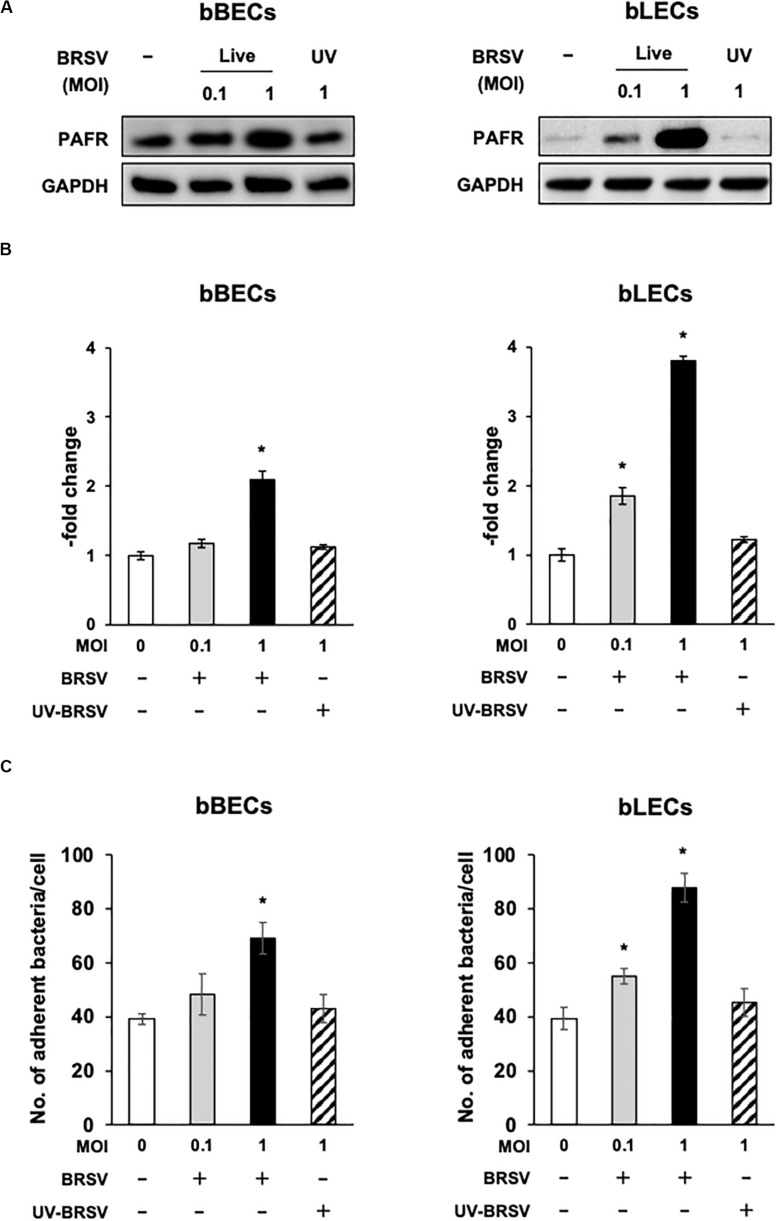
Bovine respiratory syncytial virus (BRSV) infection-mediated upregulation of platelet-activating factor receptor (PAFR) facilitates *Pasteurella multocida* (PM) adherence. **(A)** Requirement for BRSV infectivity for upregulation of PAFR in bronchus (bBECs) and lung (bLECs) epithelial cells. Cells were infected with a range multiplicity of infection (MOI) of BRSV or UV-inactivated BRSV (UV-BRSV). After 3 days, protein expression was evaluated by immunoblotting using rabbit anti-PAFR antibody. The anti-glyceraldehyde 3-phosphate dehydrogenase (GAPDH) antibody was used as the loading control. **(B)** Expression level of PAFR was calculated by normalizing the band density of PAFR by that of GAPDH. **(C)** PM adherence was enhanced by BRSV infection in a multiplicity of infection-dependent manner. PM adherence to the bBECs and bLECs nominally correlated with the level of PAFR expression. Data in **(B,C)** represent the mean ± SEM of three independent experiments using cells derived from three independent animals, each conducted in triplicate. *p < 0.05, statistical significance. Uninfected cells served as reference in **(B)**. PM adherence to uninfected cells served as reference in **(C)**.

Supporting the result of the proteomic analyses, western blotting revealed that PAFR accumulated to significantly (*p* < 0.05) higher levels in BRSV-infected bBECs and bLECs compared to uninfected cells (Figures 2A,B). Interestingly, this effect was not observed in bTECs, but nominally higher PAFR expression in bBECs infected with a higher titer of BRSV (Figures 3A,B; bBEC panels, compare MOI 0.1 and 1). A more obvious effect was observed in bLECs (Figures 3A,B; bLEC panels, compare MOI 0.1 and 1). It should be noted that this effect was viral replication-dependent (Figures 3A,B). These results suggested that BRSV enhances the expression of PAFR in cells from the lower bovine respiratory tract (bronchus and lung).

### BRSV-Induced PAFR Expression Enhances the Adherence of *Pasteurella multocida* to Bovine Lower Respiratory Epithelial Cells

Since the PAFR accumulated to significantly higher levels in bBECs and bLECs infected with BRSV (Figures 3A,B), we next tested whether enhanced PAFR expression in bovine lower respiratory epithelial cells nominally associated with increased *P. multocida* adherence. Adherence of PM to BRSV-infected bBECs and bLECs was increased depending on the virus titer used for infection (Figure 3C). The number of PM adherence (bacteria/cell) on bBECs (39.08) was significantly increased (*p* < 0.05) by infection with BRSV at MOI 1 (69.05). Furthermore, PM adherence to bLECs (39.35) was significantly increased (*p* < 0.05) by infection with BRSV at MOI 0.1 and 1 (54.98 and 87.75, respectively). PM adherence in both cells was not altered in the presence of UV-BRSV. These results suggested that PM adherence to BRSV-infected cells was MOI and virus infectivity dependent. Collectively, the magnitude of the PM adherence to the bovine lower respiratory epithelial cells infected with BRSV (Figure 3C) nominally associated with the level of PAFR expression (Figures 2, 3A–C).

### Specific Blocking and Knockdown of PAFR in BRSV-Infected Cells Prevent PM Adherence to the Bovine Lower Respiratory Epithelial Cells

**FIGURE 4:**
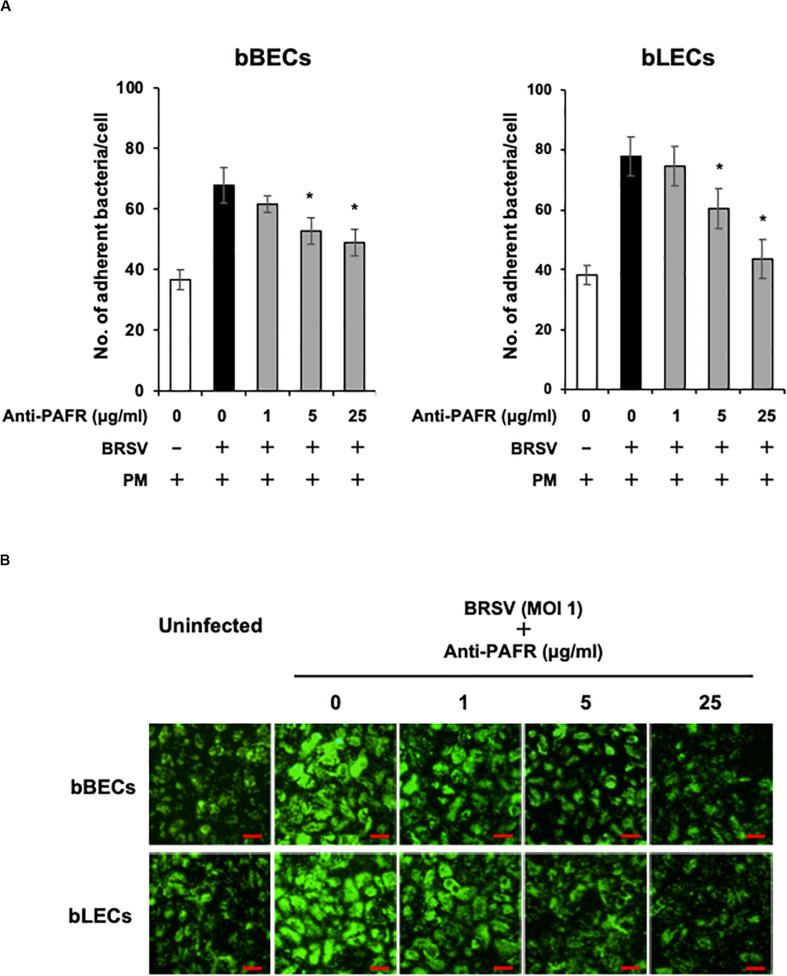
Specific blocking of platelet-activating factor receptor (PAFR) reduces *Pasteurella multocida* (PM) adherence to bovine respiratory syncytial virus (BRSV)-infected bovine respiratory epithelial cells. **(A)** Bronchus (bBECs) and lung (bLECs) epithelial cells were infected with BRSV for 3 days. The cells then were treated with a range of concentrations of the anti-PAFR antibody before being incubated with PM. The numbers of cell-associated bacteria were counted. **(B)** Observation of bBECs and bLECs under the fluorescence microscope. BRSV-infected cells treated with the anti-PAFR antibody were incubated with fluorescein isothiocyanate-labeled PM (*green*) (bar = 30 μm). The average of three independent experiments using cells derived from three independent animals conducted in triplicate is shown in **(A)**. Error bars indicate SEM; *p < 0.05, statistical significance. PM adherence to BRSV-infected cells that were not treated with antibody served as reference in **(A)**.

A blocking assay using the anti-PAFR antibody was performed to clarify the role of PAFR in enhanced PM adherence to the bovine lower respiratory epithelial cells. PM adherence to bBECs and bLECs infected with BRSV was blocked by the anti-PAFR antibody in a dose-dependent manner (Figure 4A). The number of PM adherence to BRSV-infected bBECs (67.85 bacteria/cell) was significantly decreased (*p* < 0.05) by treatment with 5 and 25 μg/ml anti-PAFR antibody (52.78 and 48.97, respectively). A similar tendency was observed in BRSV-infected bLECs (*p* < 0.05). Furthermore, fluorescence microscopic analysis demonstrated that blockade of PAFR in bBECs and bLECs led to decreased numbers of adhering FITC-labeled PM (Figure 4B). In contrast, antibody control did not affect the PM adherence to BRSV-infected cells.

**FIGURE 5:**
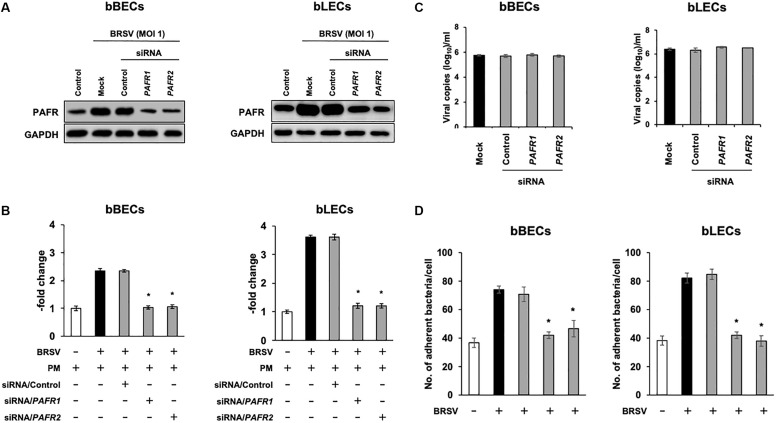
Gene-specific silencing of platelet-activating factor receptor *(PAFR)* decreases *Pasteurella multocida* (PM) adherence to bovine respiratory syncytial virus (BRSV)-infected bovine respiratory epithelial cells (BRECs). **(A)** BRECs were transfected with siRNA targeting *PAFR* (*PAFR1* and *PAFR2*). PAFR protein levels were evaluated by immunoblotting using a rabbit anti-PAFR antibody. The anti-glyceraldehyde 3-phosphate dehydrogenase (GAPDH) antibody was used as the loading control. **(B)** Expression level of PAFR was calculated by normalizing the band density of PAFR by that of GAPDH. **(C)** BRSV RNA isolated from the culture medium was analyzed by real-time quantitative reverse transcription PCR with primers targeting the *N* gene. **(D)** BRECs transfected with siRNA targeting *PAFR* (*PAFR1* and *PAFR2*) were infected with BRSV for 3 days. The cells then were incubated with PM, and the numbers of cell-associated bacteria were counted. The average of three independent experiments using cells derived from three independent animals conducted in triplicate is shown in **(B–D)**. Error bars indicate SEM; *p < 0.05, statistical significance. BRSV-infected cells that were not transfected with siRNA served as reference in **(B)**. PM adherence to BRSV-infected cells that were not transfected with siRNA served as reference in **(D)**.

We further investigated the role of PAFR binding in PM adherence to BRSV-infected bBECs and bLECs by knockdown of the *PAFR* gene using siRNA. Cells were transfected with either of two pairs of siRNAs targeting bovine *PAFR* or with a negative control siRNA and then challenged with BRSV. Both bBECs and bLECs transfected with siRNA targeting bovine *PAFR* had a significantly lower PAFR expression (*p* < 0.05) after BRSV infection (Figures 5A,B). Notably, knockdown of *PAFR* using siRNA did not affect replication of BRSV since we found comparable copy numbers of viral RNA in the culture supernatant (Figure 5C). As expected, PM adherence after BRSV infection was significantly attenuated (*p* < 0.05) in cells transfected with siRNA targeting *PAFR* (Figure 5D). PM adherence to BRSV-infected cells (70.7 bacteria/cell) was significantly decreased by knocking down the *PAFR* gene in bBECs (41.98) (*p* < 0.05). A similar tendency was observed in bLECs (84.74 on cells transfected with control siRNA, 41.28 on cells transfected with *PAFR1* siRNA, and 37.94 on cells transfected with *PAFR2* siRNA) (*p* < 0.05).

**FIGURE 6:**
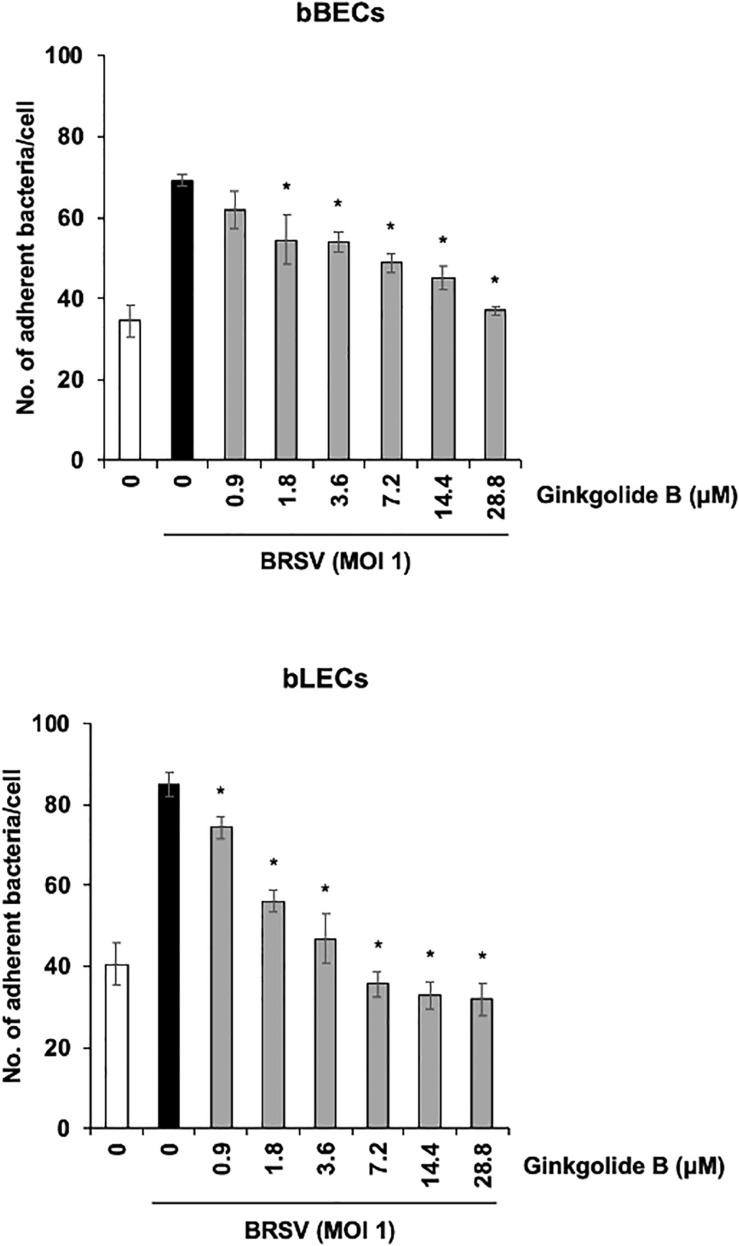
Ginkgolide B inhibits *Pasteurella multocida* (PM) adherence to bovine respiratory syncytial virus (BRSV)-infected bovine respiratory epithelial cells derived from bronchus (bBECs) and lung (bLECs) in a dose-dependent manner. BRSV-infected bBECs and bLECs were treated with a range of concentrations of ginkgolide B for 1 h and then incubated with PM. The average of three independent experiments using cells derived from three independent animals conducted in triplicate is shown. Error bars indicate SEM; *p < 0.05, statistical significance. PM adherence to BRSV-infected cells treated with dimethyl sulfoxide (DMSO) served as reference in both panels.

We further used a PAFR-specific antagonist, ginkgolide B (Chen et al., 2017), to block the function of PAFR. BRSV-infected bBECs or bLECs were incubated with increasing concentrations of ginkgolide B (0.9–28.8 μM, as a twofold dilution series) for 1 h and then exposed the cells to PM. PM adherence was evaluated by counting cell-associated PM. Ginkgolide B treatment resulted in significantly decreased PM adherence to BRSV-infected bBECs and bLECs, with adherence demonstrating a dose-dependent decrease (*p* < 0.05) (Figure 6). The number of PM adherence to BRSV-infected bBECs was significantly decreased by treatment with 1.8–28.8 μM ginkgolide B (ranging from 54.56 to 36.94 bacteria/cell) (*p* < 0.05). Similarly, ginkgolide B treatment decreased PM adherences to BRSV-infected bLECs (85.03) under 0.9–28.8 μM (ranging from 74.24 to 31.91) (*p* < 0.05).

Taken together, these data suggested that PM adherence to BRSV-infected bBECs and bLECs depends on bovine PAFR.

### Bovine PAFR Is a Major Binding Molecule for PM Adherence

**FIGURE 7:**
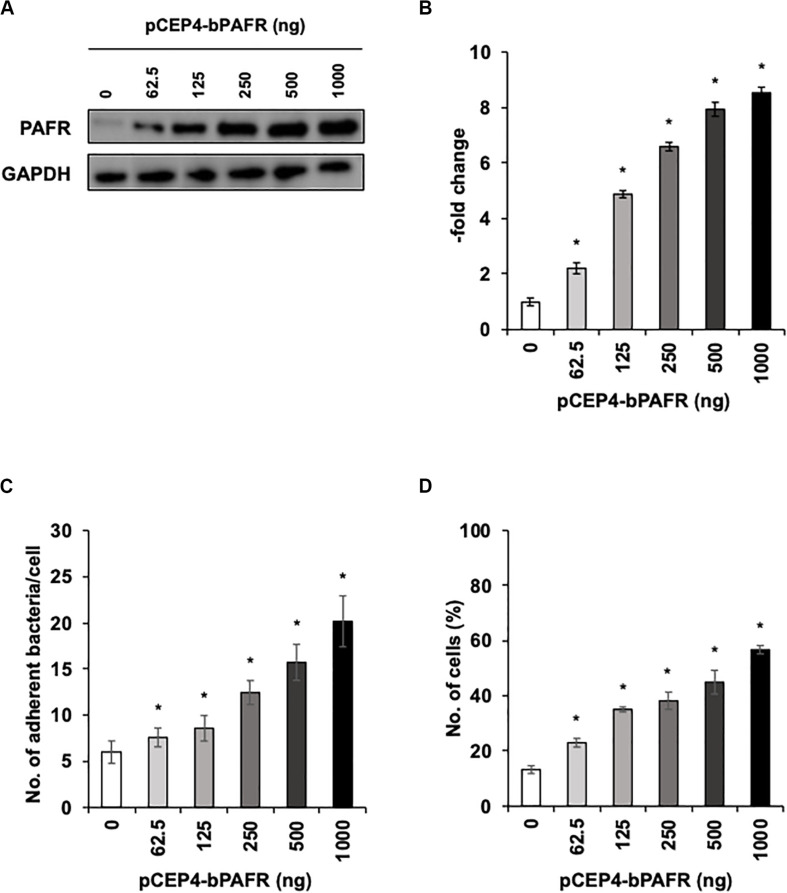
*Pasteurella multocida* (PM) adherence to HEK293T cells overexpressing bovine platelet-activating factor receptor (PAFR). **(A)** HEK293T cells were transfected with a range of amounts of pCEP4-bPAFR plasmid for 24 h. Protein expression was evaluated by immunoblotting using a rabbit anti-PAFR antibody. The anti-glyceraldehyde 3-phosphate dehydrogenase (GAPDH) antibody was used as the loading control. **(B)** Expression level of PAFR was calculated by normalizing the band density of PAFR by that of GAPDH. **(C)** Increased *Pasteurella multocida* (PM) adherence to HEK293T cells overexpressing PAFR. **(D)** HEK293T cells were transfected with a range of amounts of pCEP4-bPAFR plasmid for 24 h. Cells then were incubated with fluorescein isothiocyanate-labeled PM for 1 h. Cell-associated PM was measured by flow cytometry. The average of three independent experiments conducted in triplicate is shown in **(B–D)**. Error bars indicate SEM; *p < 0.05, statistical significance. Cells transfected with an empty vector served as reference in **(B)**. PM adherence to the cells transfected with an empty vector served as reference in **(C,D)**.

To directly test the hypothesis that bovine PAFR serves as an adherence molecule for PM, we generated a plasmid vector expressing bovine PAFR and transfected into HEK293T cells. The expression was confirmed by western blot analysis (Figures 7A,B). The result of EGFP expression demonstrated that the transfection efficiency exceeded 60% (data not shown). PM adherence assays by either counting cell-associated PM or flow-cytometric assays to detect FITC-labeled PM revealed that overexpression of bovine PAFR significantly increased PM adherence (*p* < 0.05) (Figures 7C,D). PM adherence to HEK293T cells was significantly increased by overexpression of PAFR in a dose-dependent manner (62.5–1,000 ng of plasmid DNA) as revealed by both the colony counting assay (ranging from 7.58 to 20.16 bacteria/cell) and the flow-cytometric assay (ranging from 23 to 56.7% positive cells) (*p* < 0.05).

**FIGURE 8:**
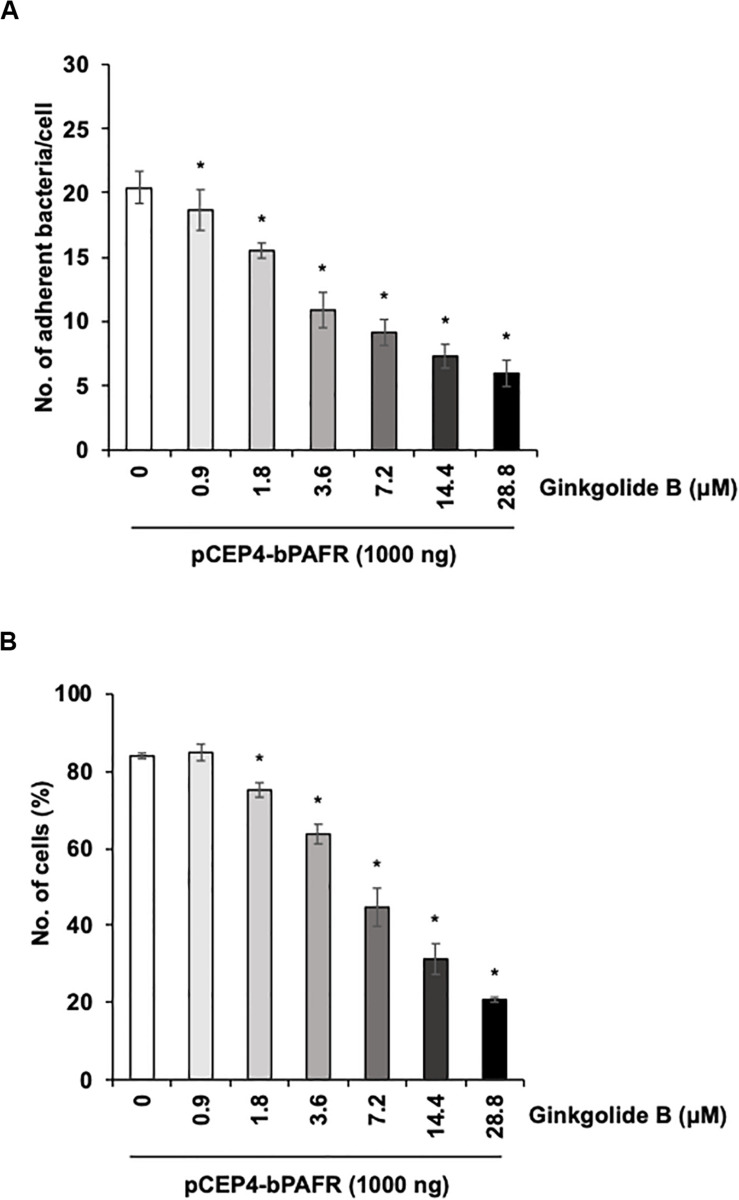
Ginkgolide B inhibits *Pasteurella multocida* (PM) adherence to HEK293T cells overexpressing bovine platelet-activating factor receptor (PAFR). **(A)** Cells overexpressing bovine PAFR were treated with a range of concentrations of ginkgolide B for 1 h. The cells then were incubated with PM for 1 h. **(B)** HEK293T cells overexpressing bovine PAFR were treated with a range of concentrations of ginkgolide B for 1 h. Cells then were incubated with fluorescein isothiocyanate-labeled PM for 1 h. Cell-associated PM was measured by flow cytometry. The average of three independent experiments conducted in triplicate is shown in **(A,B)**. Error bars indicate SEM; *p < 0.05, statistical significance. PM adherence to cells treated with DMSO served as reference in **(A,B)**.

We further tested the effect of the ginkgolide B on PM adherence to HEK293T cells expressing bovine PAFR. The results showed that ginkgolide B treatment significantly decreased PM adherence to HEK293T cells expressing bovine PAFR in a dose-dependent manner (*p* < 0.05) (Figure 8). Collectively, these data indicated that bovine PAFR is a functional binding molecule for PM adherence.

## Discussion

Bovine respiratory syncytial virus is a pathogen that is frequently found in cattle with BRDC. The results of the present study demonstrated that PAFR expression on the cell surface was upregulated by BRSV infection of bBECs and bLECs but not that of bTECs. Upregulation of PAFR in lower BRECs (bBECs and bLECs) contributed to enhanced adherence of PM. Together, our results suggested that BRSV infection-mediated upregulation of PAFR leads to augmented adherence of PM to cells of the lower respiratory tract.

Our proteomic and western blot analyses showed that BRSV infection of BRECs resulted in significant increases in PAFR accumulation in bBECs and bLECs but not in bTECs (Figures 1, 2). This phenomenon was not observed in BRECs treated with UV-BRSV, indicating that BRSV infection is necessary to trigger upregulation of PAFR (Figure 3).

Infection of cells by human rhinovirus, HRSV, coronavirus, or influenza A virus is known to increase the PAFR expression level and enhance the adhesion of *Staphylococcus aureus*, *S. pneumoniae*, and *H. influenzae* to lower respiratory epithelial cells (Avadhanula et al., 2006; Hendaus et al., 2015). Previous work has shown that PAFR expression is induced by several inflammatory factors, including interleukins-1 and -6 and tumor-necrosis factor alpha, resulting in increases in the levels of both normal cell surface-displayed and PAFR (Ishizuka et al., 2003; Garcia et al., 2010; Hamel-Côté et al., 2019). These reports suggested that regulation of bacterial adhesion-related cellular receptors is associated with the severity of pneumoniae (Wang et al., 2009; Sacco et al., 2014; Hendaus et al., 2015). Several clinical observations in cattle have suggested that viral infection of the upper respiratory tract predisposes the respiratory epithelium to bacterial adherence, permitting microorganisms to abolish physical barriers to infection in the lower respiratory tract (Larsen et al., 2001; Tjønehøj et al., 2003; Tizioto et al., 2015). Notably, BRSV facilitates secondary bacterial infection (Larsen et al., 2001; Beaudeau et al., 2010; Blodörn et al., 2015), which is associated with more severe damage in the lower respiratory tract. However, the molecular mechanism for BRDC in lower BRECs remains to be clarified.

To understand the impact of BRSV infection-associated increases in PAFR expression in lower BRECs, we performed functional analyses using molecular techniques, including specific blockade, knockdown, and overexpression of PAFR. Knockdown of *PAFR* using siRNA decreased PM adherence to the lower BRECs depending on the level of PAFR expression (Figure 5). Specific blocking of PAFR using an antibody or inhibitor also decreased PM adherence to the lower BRECs in a nominally dose-dependent manner (Figure 4). Overexpression of PAFR via a plasmid resulted in enhanced PM adherence to the lower BRECs (Figure 7). The results of these functional analyses demonstrated that PAFR is the major binding receptor for PM on bBECs and bLECs. To the best of our knowledge, this report is the first work providing evidence for the functional mechanisms underlying PM adherence to lower BRECs.

**FIGURE 9:**
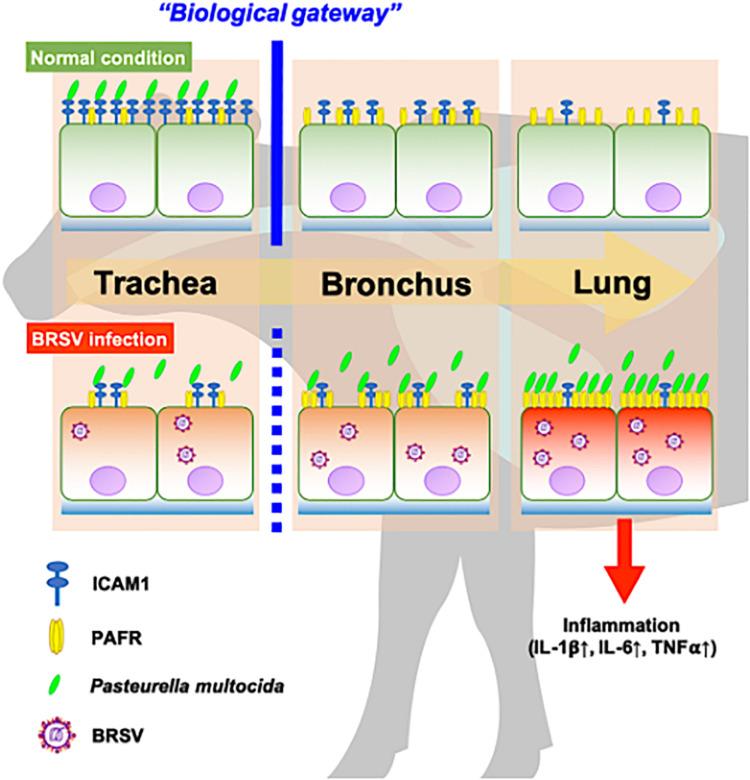
Schematic model of the synergistic effect of coinfection by bovine respiratory syncytial virus (BRSV) and *Pasteurella multocida* (PM) in cattle with severe pneumonia. Epithelial cells in the upper respiratory tract (trachea) capture PM via intercellular adhesion molecule-1 (ICAM-1) under normal conditions and serve as a biological gateway (Sudaryatma et al., 2020). BRSV infection abrogates this gateway function by downregulating ICAM-1 on the epithelial cells of the upper respiratory tract and upregulating the platelet-activating factor receptor (PAFR) on the epithelial cells of the lower respiratory tract. This change permits PM to invade the lower respiratory tract, stimulating inflammation in the lung (Sudaryatma et al., 2019).

In recent work (Sudaryatma et al., 2020), we have obtained evidence suggesting that the upper respiratory tract is responsible for inhibiting PM invasion of the lower respiratory tract. This inhibition is accomplished by trapping PM via ICAM-1, an adherence molecule that is displayed on the cellular surface under normal conditions. By capturing microbes, ICAM-1 displayed by cells of the upper respiratory tract acts as a “gateway” that impedes bacterial invasion of the lower respiratory tract (Sudaryatma et al., 2020). BRSV infection causes malfunction of ICAM-1 in the bovine upper respiratory tract, allowing bacterial invasion of the lower respiratory tract (Sudaryatma et al., 2020). The results of the present study demonstrated that BRSV infection increased PAFR expression in epithelial cells derived from the lower respiratory tract. We further demonstrated that PM adherence to cells of the lower respiratory tract was enhanced by upregulation of PAFR. Overall, these findings highlight the importance of the synergistic effect of BRSV infection on severe pneumonia in cattle (Figure 9). Effective interventions to treat this complex interaction are required to improve cattle heath and the economic gains of farmers.

## Data Availability Statement

The original contributions presented in the study are publicly available. This data can be found in jPOSTrepo (Japan proteome standard repository) under the ID: JPST000862 and/or http://www.proteomexchange.org/, under the ID: PXD019509.

## Author Contributions

PS, AS, and TO designed the study. PS and WF performed the experiments. PS drafted the manuscript. AS, HM, MK, EM, and TO revised the manuscript. AS and TO supervised the experimental work. MK provided the cattle respiratory organs. HM contributed the reagents and materials. All authors have read, commented on, and approved the manuscript.

## Conflict of Interest

The authors declare that the research was conducted in the absence of any commercial or financial relationships that could be construed as a potential conflict of interest.

## Footnotes

1 www.interactivenn.net
